# Foundation model for screening severe mitral regurgitation and severe aortic stenosis from coronary angiograms

**DOI:** 10.1186/s42492-026-00221-5

**Published:** 2026-06-17

**Authors:** Yingqian Zhang, Zhiming Shao, Zechen Wei, Changfu Liu, Zeyu Sun, Liangliang Liu, Ran Xin, Yong Ge, Chen Wang, Guodong Ding, Lei Gao, Liwei Zhang, Hui Hui, Jie Tian, Yundai Chen

**Affiliations:** 1 Senior Department of Cardiology, Chinese PLA General Hospital, Beijing, 100048 China; 2 Beijing Key Laboratory of Molecular Imaging Technology Research and Translation, Institute of Automation, Chinese Academy of Sciences, Beijing, 100190 China; 3 School of Artificial Intelligence, University of Chinese Academy of Sciences, Beijing, 100190 China; 4 Department of Biomedical Engineering, Hong Kong Polytechnic University, Hong Kong, 999077 China; 5 School of Medicine, Nankai University, Tianjin, 300071 China; 6 Medical School of Chinese PLA, Chinese PLA General Hospital, Beijing, 100853 China; 7 Bo Ai Hospital of Huanghua City, Cangzhou, 061100 Hebei China; 8 Qingyun County People’s Hospital, Dezhou, 253700 Shandong China; 9 Qixia People’s Hospital of Yantai City, Yantai, 265300 Shandong China; 10 School of Engineering Medicine and School of Biological Science and Medical Engineering, Beihang University, Beijing, 100191 China

**Keywords:** Coronary angiography, Aortic stenosis, Mitral regurgitation, Foundation model

## Abstract

Coronary heart disease (CAD) is the leading cause of death worldwide, and coronary angiography (CAG) serves as the gold standard for its assessment. Valvular heart diseases, such as severe aortic stenosis (AS) and severe mitral regurgitation (MR), frequently coexist with CAD yet are often underdiagnosed. Opportunistic screening for these conditions at the time of CAG could influence therapeutic strategies and improve prognosis. This study developed and validated a foundation model for the automated screening of severe AS and severe MR from CAG videos. The study presents CAGFound, a video-based foundation model that was self-supervised pre-trained on CAG sequences from seven medical centers and subsequently adapted to two downstream tasks: screening for severe AS and severe MR. Two internal and external validation datasets were retrospectively enrolled from the First Medical Center and the Sixth Medical Center of Chinese PLA General Hospital, respectively. A total of 117,383 unlabeled CAG sequences were used to build CAGFound. For the detection of severe AS, CAGFound achieved an area under the receiver operating characteristic curve (AUROC) of 0.932 (sensitivity 0.767, specificity 0.921) on the internal test dataset and maintained robust performance on the external validation dataset, with an AUROC of 0.879 (sensitivity 0.800, specificity 0.955). For the detection of severe MR, the model demonstrated an AUROC of 0.933 (sensitivity 0.738, specificity 0.938) on the internal dataset and an AUROC of 0.896 (sensitivity 0.754, specificity 0.855) on the external cohort. The performance of CAGFound was also compared with other video-based foundation models, VideoMAEv2 and Video Swin. CAGFound achieved the highest AUROC and demonstrated the best calibration performance (Brier score 0.122, R^2^ 0.478) compared with VideoMAEv2 (Brier score 0.159, R^2^ 0.306) and Video Swin (Brier score 0.162, R^2^ 0.306). CAGFound enables accurate, automated screening for severe AS and severe MR during CAG. It has the potential to increase detection rates, facilitate timely clinical referral, and improve prognosis without requiring additional contrast administration or procedures.

**Supplementary Information:**

The online version contains supplementary material available at 10.1186/s42492-026-00221-5.

## Introduction

Valvular heart disease (VHD) is a major contributor to heart failure, reduced quality of life, and premature mortality [1, 2]. Mitral regurgitation (MR) and aortic stenosis (AS) are the two most prevalent forms of VHD [3–5]. Coexisting coronary heart disease (CAD) is common in patients with severe AS, affecting more than 50% of individuals aged 70 years and older [6, 7]. CAD is also a frequent cause of severe MR [8]. Despite their high prevalence and clinical significance, more than half of patients with AS or MR remain undiagnosed [9–11]. Patients with severe AS often present with chest pain that may mimic CAD, further complicating timely diagnosis [12, 13]. Coronary angiography (CAG), which is performed to evaluate CAD, may contain subvisual information related to severe AS and severe MR that is not utilized in current clinical practice. Early identification of severe AS and severe MR in patients undergoing CAG is crucial to reduce misdiagnosis and guide appropriate treatment strategies. A novel method to proactively identify life-threatening VHDs using already acquired CAG imaging data could improve the efficiency of medical resource utilization without imposing additional burdens on patients or prolonging procedure time.

In addition to illustrating coronary anatomy associated with CAD, CAG videos provide valuable insights into cardiac remodeling, arrhythmia, and epicardial arterial motion [14, 15]. These characteristics differ between patients with and without VHD. Artificial intelligence has demonstrated strong capability in real-time coronary artery segmentation, automated assessment of cardiac systolic function, and quantitative angiographic analysis directly from CAG [16–18]. A foundation model leverages large amounts of unlabeled data to learn general-purpose features. Following the pretraining stage, it is fine-tuned for specific downstream tasks [19]. Such models have outperformed supervised learning-based transfer learning approaches and have enhanced the robustness of lung cancer screening and echocardiographic interpretation [20, 21]. The study hypothesized that severe AS and severe MR could be detected from CAG using a video-based foundation model. Accordingly, the study developed CAGFound, a foundation model pretrained on large volumes of unlabeled angiographic videos from seven medical centers. The results demonstrate its ability to function as an automated, real-time screening tool for severe AS and MR, and the model was externally validated using an independent dataset.

## Methods

### Datasets for developing CAGFound

CAGFound was pretrained on a dataset comprising 117,383 unlabeled CAG videos acquired from 12,435 patients. The CAG videos were retrospectively collected from the First Medical Center of Chinese PLA General Hospital, the Fourth Medical Center of Chinese PLA General Hospital, the Sixth Medical Center of Chinese PLA General Hospital, Qilu Hospital of Shandong University, Boai Hospital of Hebei Province, Qixia City People’s Hospital of Shandong Province, and Qingyun County People’s Hospital of Shandong Province. All eligible participants provided written informed consent. The protocol was approved by the Ethics Committee of the Chinese PLA General Hospital (No. S2024-692-01) and was conducted in accordance with the Declaration of Helsinki. The inclusion criteria were as follows: patients aged more than 18 years and patients who underwent standard invasive CAG. The exclusion criteria were as follows: poor image quality and CAG videos lacking visualization of the left coronary artery.

### Datasets for severe AS diagnosis

The internal test dataset for severe AS was retrospectively enrolled from the First Medical Center of Chinese PLA General Hospital between 2013 and 2025. It consisted of 358 CAG sequences, including 119 patients with severe AS and 239 controls. Details of the datasets, including patient demographics and the absolute time interval between angiography and transthoracic echocardiography (TTE), are listed in Table S1 in Additional File 1. The external validation dataset was obtained from the Sixth Medical Center of Chinese PLA General Hospital between 2021 and 2025. It consisted of 134 CAG sequences, including 45 patients with severe AS and 89 controls. The internal and external test datasets included patients who underwent CAG and TTE within one month before or after the angiographic procedure. The TTE performed closest to the date of angiography was used as the reference standard [18]. For TTE, data on severe AS were extracted from the final echocardiographer diagnostic reports. Echocardiographers followed the recommendations of the American Society of Echocardiography for the evaluation of valvular regurgitation and stenosis and used an integrated approach incorporating qualitative, semiquantitative, and quantitative parameters [22]. All CAG videos were saved in DICOM format, and left coronary angiographic sequences were extracted for model development. In CAG, left cranial or right cranial projection views were used for model construction and testing. Two cardiologists performed medical record review to collect patient characteristics. Additional details are available in Tables S1 and S2 in Additional File 1.

### Dataset for severe MR diagnosis

The internal test dataset for severe MR was retrospectively enrolled from the First Medical Center of Chinese PLA General Hospital between 2013 and 2025. It consisted of 848 CAG sequences, including 280 patients with severe MR and 568 controls. Details of the severe MR dataset are listed in Table S3 in Additional File 1. The external validation dataset for severe MR was obtained from the Sixth Medical Center of Chinese PLA General Hospital between 2021 and 2025. It consisted of 207 CAG sequences, including 69 patients with severe MR and 138 controls. The internal and external datasets included patients who underwent CAG and TTE within one month before or after angiography. The TTE performed closest to the date of angiography was used as the reference standard [18]. For TTE, data on severe MR were extracted from the final echocardiographer diagnostic reports. All CAG videos were saved in DICOM format, and left coronary angiographic sequences were extracted for model development. In CAG, left cranial or right cranial projection views were used for model construction and testing. Two cardiologists performed medical record review to collect patient characteristics. All eligible participants provided written informed consent. Additional details are available in Tables S3 and S4 in Additional File 1.

### Data processing and augmentation for self-supervised learning

Each CAG video was resized to 224 × 224 pixels using cubic interpolation. For model input, 16 frames were uniformly sampled at 3-frame intervals from each video. If a video contained fewer than the required number of frames (i.e., fewer than 3 × 16 frames), the last available frame was repeated to pad the sequence to the required length.

### CAGFound architecture and implementation

**Fig. 1:**
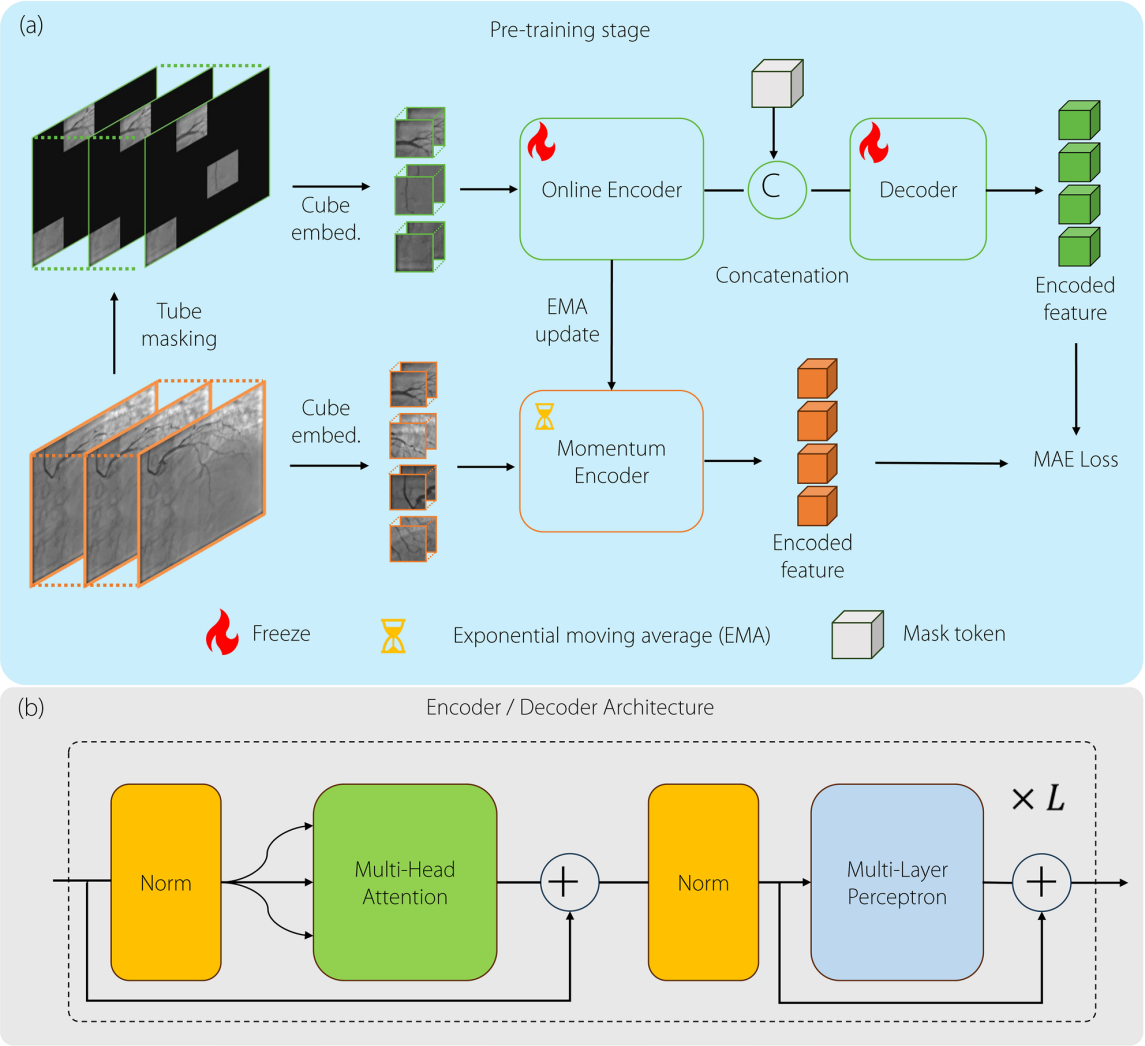
Architecture of CAGFound. **a** Pretraining stage: The framework applies tube masking to input coronary angiography videos and utilizes an online encoder alongside a momentum encoder (updated via exponential moving average) for feature extraction. The decoder reconstructs features from the concatenated online embeddings and mask tokens, which are optimized against the momentum features using mean absolute error loss; **b** Encoder-decoder architecture: Detailed view of the Transformer-based blocks consisting of normalization, multi-head attention, and multilayer perceptron layers, repeated L times

A specific configuration of the video joint-embedding predictive architecture was adopted [23], which comprises an online encoder, a momentum encoder, and a projector. The architectural details are illustrated in Fig. 1. The online encoder was based on the base vision Transformer (ViT-base) [24] with 12 Transformer blocks and an embedding dimension of 768. It processed unmasked video cubes (cube size 2 × 16 × 16 pixels) and mapped them to a 768-dimensional feature vector. These features were then refined through 12 Transformer blocks–each consisting of multi-head self-attention and a multilayer perceptron (MLP) –to produce high-level representations. The momentum encoder mirrored the architecture of the online encoder but maintained an exponential moving average (EMA) of its weights and did not receive gradient updates during training. Its role was to provide stable target embeddings for the predictive task. The projector was implemented as a lightweight vision Transformer (ViT-Small) with 4 Transformer blocks and an embedding dimension of 384. Given the high-level features from the online encoder, the projector predicted the embeddings of masked regions, which were compared with the corresponding outputs from the momentum encoder.

During training, the model was optimized to predict the momentum encoder’s representations of highly masked video inputs, using a mask ratio of 0.9. Training was conducted with a batch size of 128 for 200 epochs. The first 20 epochs were dedicated to a learning rate warm-up schedule, during which the learning rate was linearly increased from 0 to 1 × 10^−3^. The checkpoint from the final epoch was retained for downstream task adaptation.

### Adaptation to downstream tasks

For downstream task adaptation, only the online encoder (ViT-base) of the foundation model was retained. The online encoder generated high-level feature representations from CAG images. A MLP took these features as input and output the probability of each category (normal or abnormal). The number of categories (e.g., 2) determined the number of neurons in the final layer of the MLP. To improve generalization and prevent overfitting, label smoothing was applied during training. This technique regularizes the model by softening the ground-truth labels, thereby mitigating overconfidence in predictions. The training objective was to generate categorical outputs consistent with the reference labels. The batch size was 16. The total number of training epochs was 10, with the first 2 epochs used for learning rate warm-up (from 0 to 7 × 10^−4^), followed by a cosine annealing schedule (from 7 × 10^−4^ to 1 × 10^−6^ over the remaining 8 epochs). After each epoch, two cycles of 5-fold cross-validation were performed to comprehensively evaluate the downstream transferability of the pretrained model [19]. The pretrained model was fine-tuned on the internal dataset and subsequently evaluated on the external validation datasets.

### Computational resources

Self-supervised learning (SSL) typically benefits from large batch sizes during training to enhance contextual representation learning, which requires powerful graphics processing units for computation. One NVIDIA Tesla A800 (80 GB) was used, and model development required approximately 5 days. For fine-tuning CAGFound on downstream tasks, computational resources comparable to those used for pretraining were allocated. Fine-tuning required approximately 1 min per 100 images.

### Evaluation and statistical analysis

The area under the receiver operating characteristic curve (AUROC), sensitivity, specificity, accuracy, positive predictive value (PPV), and negative predictive value (NPV) were calculated to evaluate model performance. The F1 score, defined as the harmonic mean of precision and recall, provides a balanced performance measure. Cohen’s kappa coefficient was used to assess agreement between predicted and true labels while correcting for chance agreement. For each metric, 95%CIs were calculated. The CI for AUROC was estimated using the DeLong method, whereas CIs for proportion-based metrics (accuracy, sensitivity, specificity, PPV, and NPV) were computed using the Wilson binomial method. For the F1 score and Cohen’s kappa, 95%CIs were obtained via nonparametric bootstrap resampling with 1000 repetitions. All statistical analyses were performed using Python (version 3.10).

Calibration curves were constructed by grouping predicted probabilities into 10 equal-frequency bins and plotting the mean predicted probability against the observed event rate within each bin. Decision curve analysis (DCA) was employed to evaluate the clinical utility of each model by quantifying the net clinical benefit across the threshold probability range of 0–100%. The analysis utilized specialized packages, including rms (for calibration statistics) and rmda (for DCA), in R software (version 4.3.0).

## Results

### Patient characteristics

**Fig. 2:**
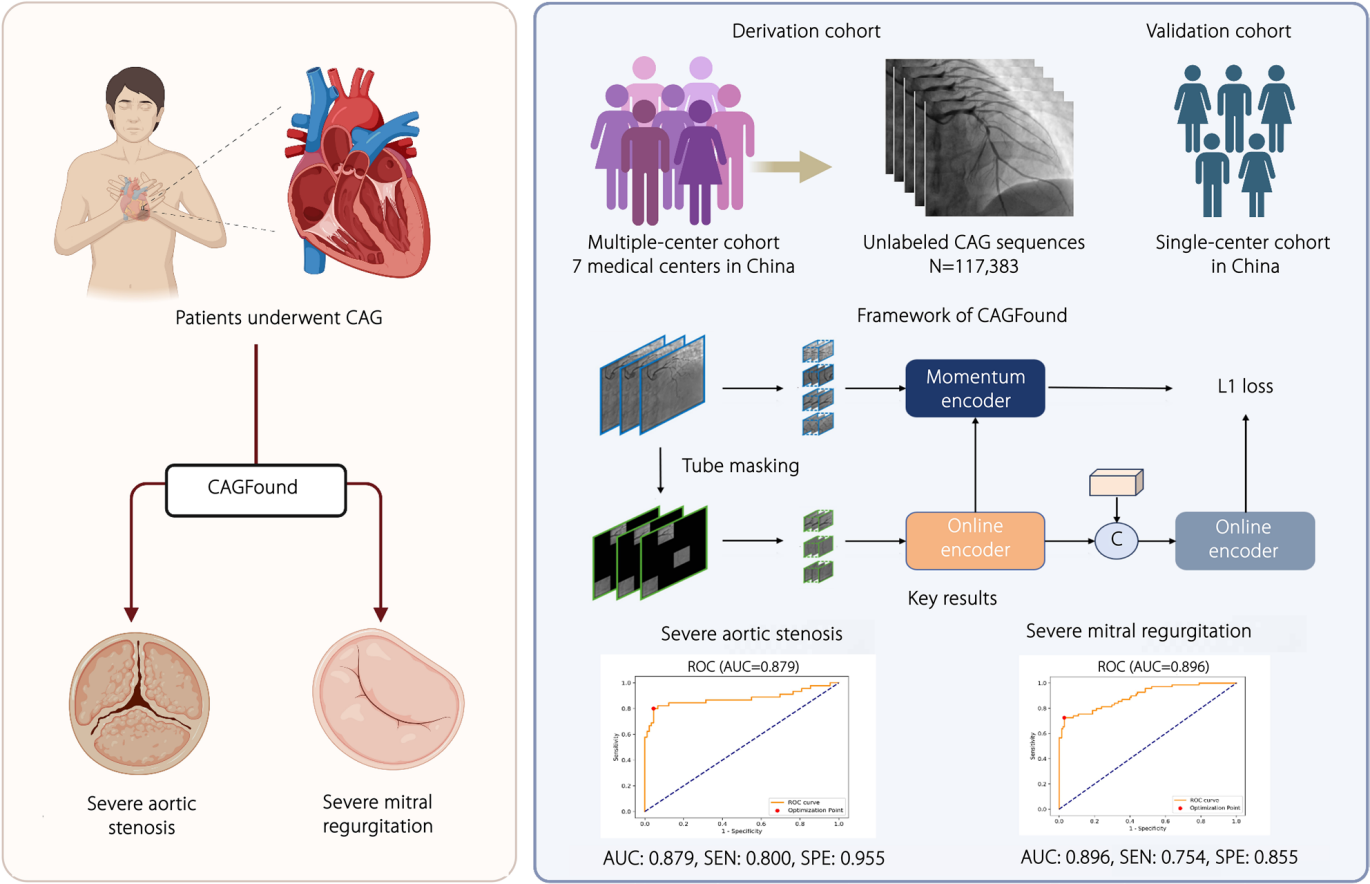
CAGFound workflow. CAGFound is a video-based foundation model trained on 117,383 unlabeled coronary angiography (CAG) videos from seven medical centers. It was adapted to two downstream tasks: detection of severe AS and severe MR. Generalizability was evaluated using an external dataset from a separate medical center. The AUROC for severe AS and severe MR demonstrates robust performance in external validation. CAG: Coronary angiography; AS: Aortic stenosis; MR: Mitral regurgitation; AUROC: Area under the receiver operating characteristic curve

CAGFound is a video-based model trained on 117,383 unlabeled CAG videos from 12,435 patients across seven medical centers (Fig. 2). The mean patient age was 61.65 ± 10.58 years, and 8903 patients were male (71.60%). In the internal severe AS dataset (*n* = 358), 119 patients had severe AS. The mean age was 68.42 ± 9.86 years, and 68 patients were male (57.14%). The mean interval between angiography and TTE was 5.52 ± 5.26 days, with most TTE examinations performed before angiography. Among the 239 patients without severe AS, the majority presented with acute coronary syndromes, including 2.93% with non-ST-segment elevation myocardial infarction (NSTEMI), 7.95% with ST-segment elevation myocardial infarction (STEMI), and 81.95% with unstable angina. These patients were less likely to be female (28.03%, *P* = 0.007) and more likely to have hypertension (75.73% *vs* 63.03%, *P* < 0.001). The mean interval between angiography and TTE in this group was 2.81 ± 3.33 days. Detailed characteristics are provided in Supplementary Table S1. The internal severe MR test dataset consisted of 848 CAG sequences from 280 patients with severe MR and 568 controls. Patients with severe MR were older than those without severe MR (mean age 65.34 ± 8.42 *vs* 61.70 ± 10.12 years, *P* < 0.001) and included a lower proportion of males (48.93% *vs* 77.11%, *P* < 0.001). Most patients without severe MR presented with acute coronary syndromes (4.05% NSTEMI, 1.76% STEMI, and 88.20% unstable angina). The interval between TTE and CAG was longer in patients with severe MR (5.57 ± 5.76 *vs* 3.57 ± 3.85 days, *P* < 0.001). Most patients underwent TTE before CAG (Table S2 in Additional File 1).

The external severe AS dataset consisted of 45 patients with severe AS and 89 controls. Patients with severe AS were older than controls (67.27 ± 10.43 *vs* 62.01 ± 10.37 years, *P* = 0.007). A higher proportion of female patients was observed in the severe AS group. Body mass index was lower in the severe AS group. The mean absolute interval between TTE and CAG was around 3 days in both groups (Table S3 in Additional File 1). The external severe MR test dataset consisted of 69 patients with severe MR and 138 controls. Patients with severe MR were older than controls (63.87 ± 8.32 *vs* 60.86 ± 10.77 years, *P* = 0.028). They were more likely to be female (49.28% *vs* 26.09%, *P* = 0.001) and less likely to have hypertension (49.28% *vs* 68.12%, *P* =0.009). The absolute interval between TTE and CAG was longer in patients with severe MR (5.49 ± 5.71 *vs* 3.67 ± 4.14 days, *P* = 0.010). Approximately one-quarter of patients with severe MR underwent TTE after CAG (Table S4 in Additional File 1).

### Evaluation of CAGFound performance

The diagnostic performance of CAGFound was evaluated on two classification tasks: detection of severe AS and severe MR. In the internal severe AS dataset, CAGFound demonstrated a strong ability to discriminate patients with severe AS. In 5-fold cross-validation, the model achieved a mean AUROC of 0.932, with a mean sensitivity of 0.767 and a mean specificity of 0.921. The mean PPV was 0.856, and the mean NPV was 0.878. The model attained a mean accuracy of 0.866, an F1 score of 0.803, and a Cohen’s kappa coefficient of 0.702. In the internal severe MR dataset, CAGFound also demonstrated robust performance in detecting severe MR. The mean AUROC was 0.933. The mean sensitivity was 0.738, and the mean specificity was 0.938. The mean PPV and NPV were 0.863 and 0.879, respectively. The model achieved a mean accuracy of 0.871, an F1 score of 0.793, and a Cohen’s kappa coefficient of 0.700. The performance metrics for each fold are presented in Table 1.

**Table 1:** Performance of CAGFound in identifying severe AS and severe MR in the internal test set

Fold	AUROC (95%CI)	Sensitivity (95%CI)	Specificity (95%CI)	PPV (95%CI)	NPV (95%CI)	Accuracy (95%CI)	F1	Kappa
**Severe AS internal test set**								
0	0.934 (0.867–1.000)	0.815 (0.633–0.918)	0.875 (0.753–0.941)	0.786 (0.605–0.898)	0.894 (0.774–0.954)	0.853 (0.756–0.916)	0.800	0.684
1	0.926 (0.854–0.998)	0.667 (0.478–0.814)	1.000 (0.926–1.000)	1.000 (0.824–1.000)	0.842 (0.726–0.915)	0.880 (0.787–0.936)	0.800	0.719
2	0.966 (0.917–1.000)	0.815 (0.633–0.918)	0.938 (0.832–0.979)	0.880 (0.700–0.958)	0.900 (0.786–0.957)	0.893 (0.803–0.945)	0.846	0.765
3	0.937 (0.869–1.000)	0.731 (0.539–0.863)	0.938 (0.832–0.979)	0.864 (0.667–0.953)	0.865 (0.747–0.933)	0.865 (0.769–0.925)	0.792	0.693
4	0.896 (0.810–0.982)	0.808 (0.621–0.915)	0.854 (0.728–0.928)	0.750 (0.566–0.873)	0.891 (0.770–0.953)	0.838 (0.738–0.905)	0.778	0.650
**Severe MR internal test set**								
0	0.946 (0.904–0.988)	0.776 (0.653–0.864)	0.920 (0.856–0.958)	0.833 (0.713–0.910)	0.889 (0.819–0.934)	0.871 (0.813–0.913)	0.804	0.708
1	0.946 (0.904–0.988)	0.759 (0.635–0.850)	0.938 (0.878–0.970)	0.863 (0.743–0.932)	0.883 (0.814–0.929)	0.877 (0.820–0.918)	0.807	0.718
2	0.953 (0.914–0.992)	0.754 (0.629–0.848)	0.956 (0.901–0.981)	0.896 (0.778–0.955)	0.886 (0.818–0.931)	0.889 (0.833–0.928)	0.819	0.740
3	0.917 (0.865–0.969)	0.789 (0.667–0.875)	0.904 (0.835–0.945)	0.804 (0.682–0.887)	0.896 (0.826–0.939)	0.865 (0.806–0.909)	0.796	0.696
4	0.903 (0.847–0.958)	0.614 (0.484–0.729)	0.974 (0.925–0.991)	0.921 (0.792–0.973)	0.842 (0.726–0.915)	0.854 (0.793–0.899)	0.737	0.641

Abbreviations: AS, aortic stenosis; MR, mitral regurgitation; AUROC, area under the receiver operating characteristic curve; PPV, positive predictive value; NPV, negative predictive value

### Generalization to a different hospital

**Fig. 3:**
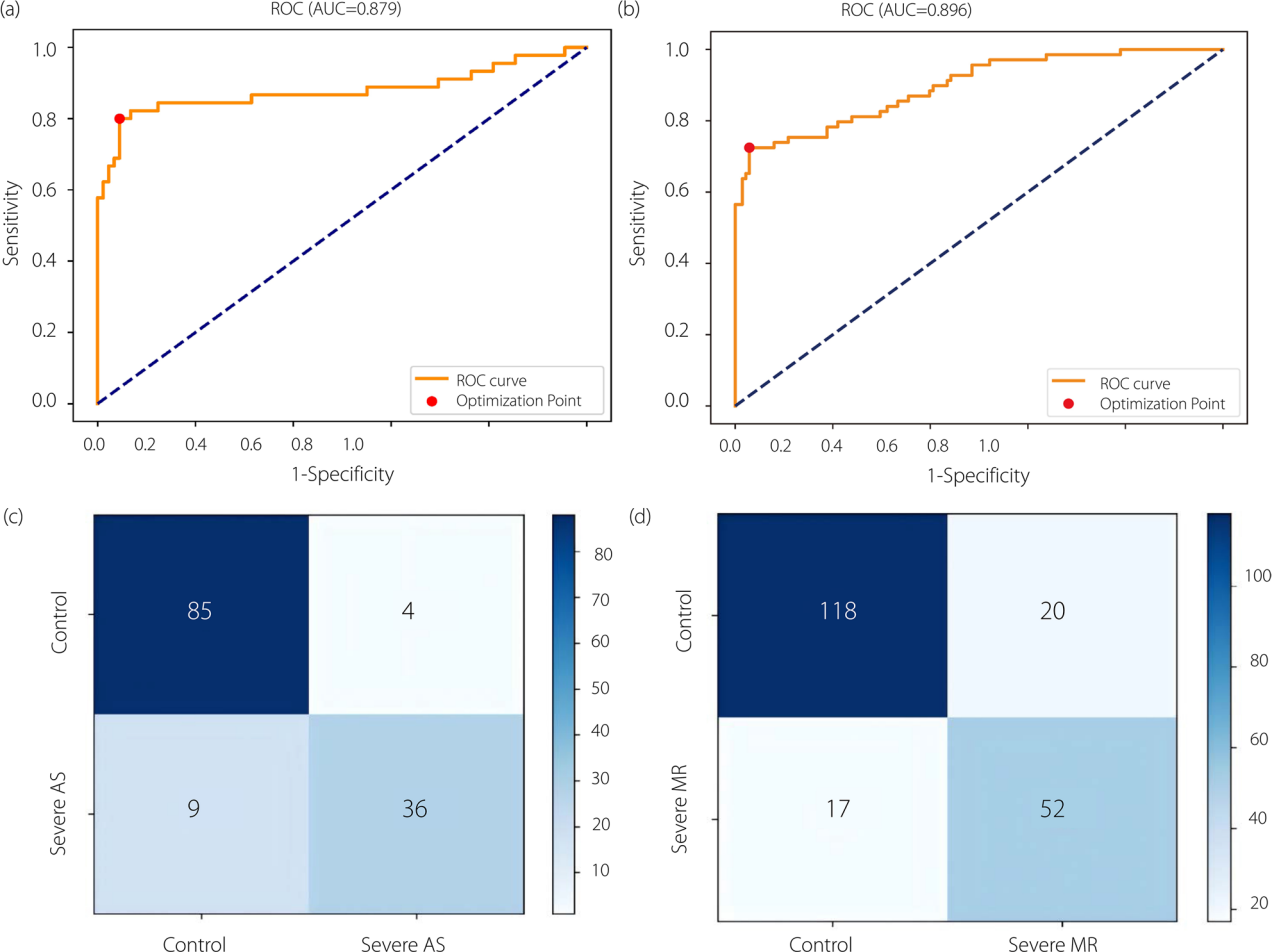
CAGFound performance in external datasets. **a** Receiver operating characteristic curve for the diagnosis of severe AS; **b** Receiver operating characteristic curve for the diagnosis of severe MR; **c** Confusion matrix for severe AS diagnosis; **d** Confusion matrix for severe MR diagnosis. AS: Aortic stenosis; MR: Mitral regurgitation

In the external validation dataset for severe AS, CAGFound demonstrated robust discriminative performance, achieving an AUROC of 0.879 (95%CI: 0.809–0.948) (Fig. 3a). The sensitivity was 0.800 (95%CI: 0.662–0.891), and the specificity was 0.955 (95%CI: 0.890–0.982). The PPV was 0.900 (95%CI: 0.769–0.960), and the NPV was 0.904 (95%CI: 0.827–0.949). The accuracy was 0.903 (95%CI: 0.841–0.942). The F1 score and Cohen’s kappa coefficient were 0.847 and 0.776, respectively, in the external validation dataset, further supporting the model’s generalizability.

In the external validation dataset for severe MR, CAGFound accurately identified severe MR, achieving an AUROC of 0.896 (95%CI: 0.844–0.948) (Fig. 3b). The sensitivity was 0.754 (95%CI: 0.640–0.840), and the specificity was 0.855 (95%CI: 0.787–0.904). CAGFound achieved a PPV of 0.722 (95%CI: 0.610–0.812) and an NPV of 0.874 (95%CI: 0.808–0.920). The overall accuracy was 0.821 (95%CI: 0.763–0.867). The F1 score and Cohen’s kappa coefficient were 0.738 and 0.602, respectively, indicating consistent external performance.

### Comparison with other video-based foundation models

**Fig. 4:**
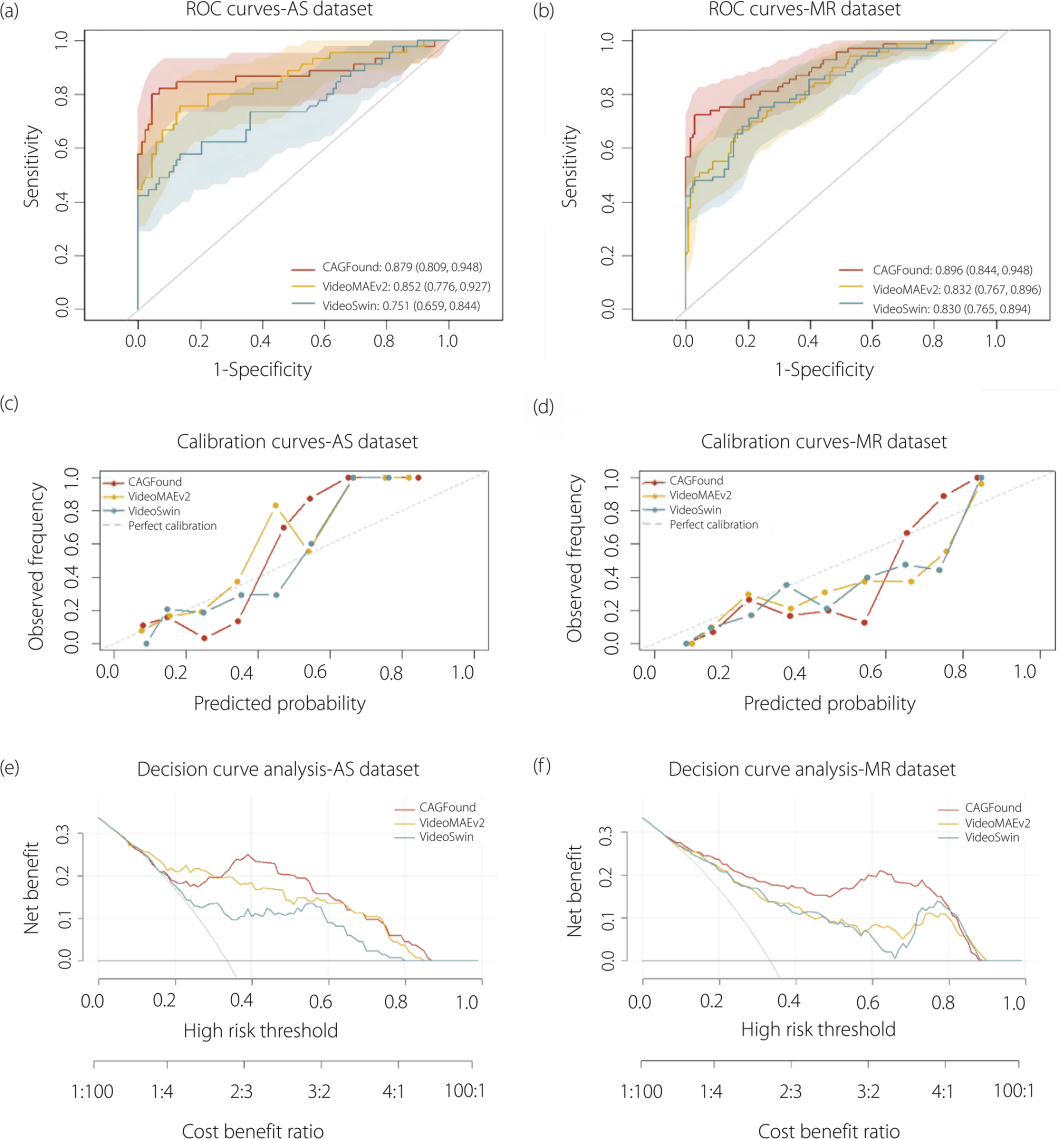
Comparison of different models for severe AS and severe MR diagnosis. **a** Receiver operating characteristic curve comparing the diagnostic performance of CAGFound, VideoMAEv2, and Video Swin for severe AS; **b** Receiver operating characteristic curve comparing the diagnostic performance of CAGFound, VideoMAEv2, and Video Swin for severe MR; **c** Calibration analysis of the three models for the detection of severe AS; **d** Calibration analysis of the three models for the detection of severe MR; **e** DCA of the three models for the detection of severe AS; **f** DCA of the three models for the detection of severe MR. AS: Aortic stenosis; MR: Mitral regurgitation; DCA: Decision curve analysis

The performance of CAGFound was compared with other video-based foundation models, VideoMAEv2 [25] and Video Swin [26]. For severe AS diagnosis, CAGFound achieved an AUROC of 0.879 (95%CI: 0.809–0.948). The AUROC of VideoMAEv2 for severe AS detection was 0.852 (95%CI: 0.776–0.927), and that of Video Swin was 0.751 (95%CI: 0.659–0.844) (Fig. 4a). For severe MR detection, CAGFound again demonstrated superior performance, achieving an AUROC of 0.896 (95%CI: 0.844–0.948), compared with 0.832 (95%CI: 0.767–0.896) for VideoMAEv2 and 0.830 (95%CI: 0.765–0.894) for Video Swin (Fig. 4b). Calibration analysis further confirmed the robustness of CAGFound. In the severe AS cohort, CAGFound achieved the optimal calibration profile, with the lowest Brier score (0.130) and the highest R^2^ value (0.481), outperforming VideoMAEv2 (Brier score 0.137, R^2^ 0.427) and Video Swin (Brier score 0.176, R^2^ 0.222) (Fig. 4c). This performance advantage was consistently observed in the severe MR cohort, in which CAGFound again demonstrated the best calibration performance (Brier score 0.122, R^2^ 0.478) compared with VideoMAEv2 (Brier score 0.159, R^2^ 0.306) and Video Swin (Brier score 0.162, R^2^ 0.306) (Fig. 4d).

DCA demonstrated a consistent net clinical benefit of CAGFound across both severe AS and severe MR datasets. CAGFound provided greater net clinical benefit than the comparator models at decision thresholds exceeding 10%, and this advantage was maintained throughout clinically relevant ranges (15%–80% for MR and 40%–60% for AS) (Figs. 4e, f). CAGFound outperformed VideoMAEv2 and Video Swin in both downstream tasks. All quantitative results are presented in Tables S5 and S6 in Additional File 1.

### Model interpretability and visualization

**Fig. 5:**
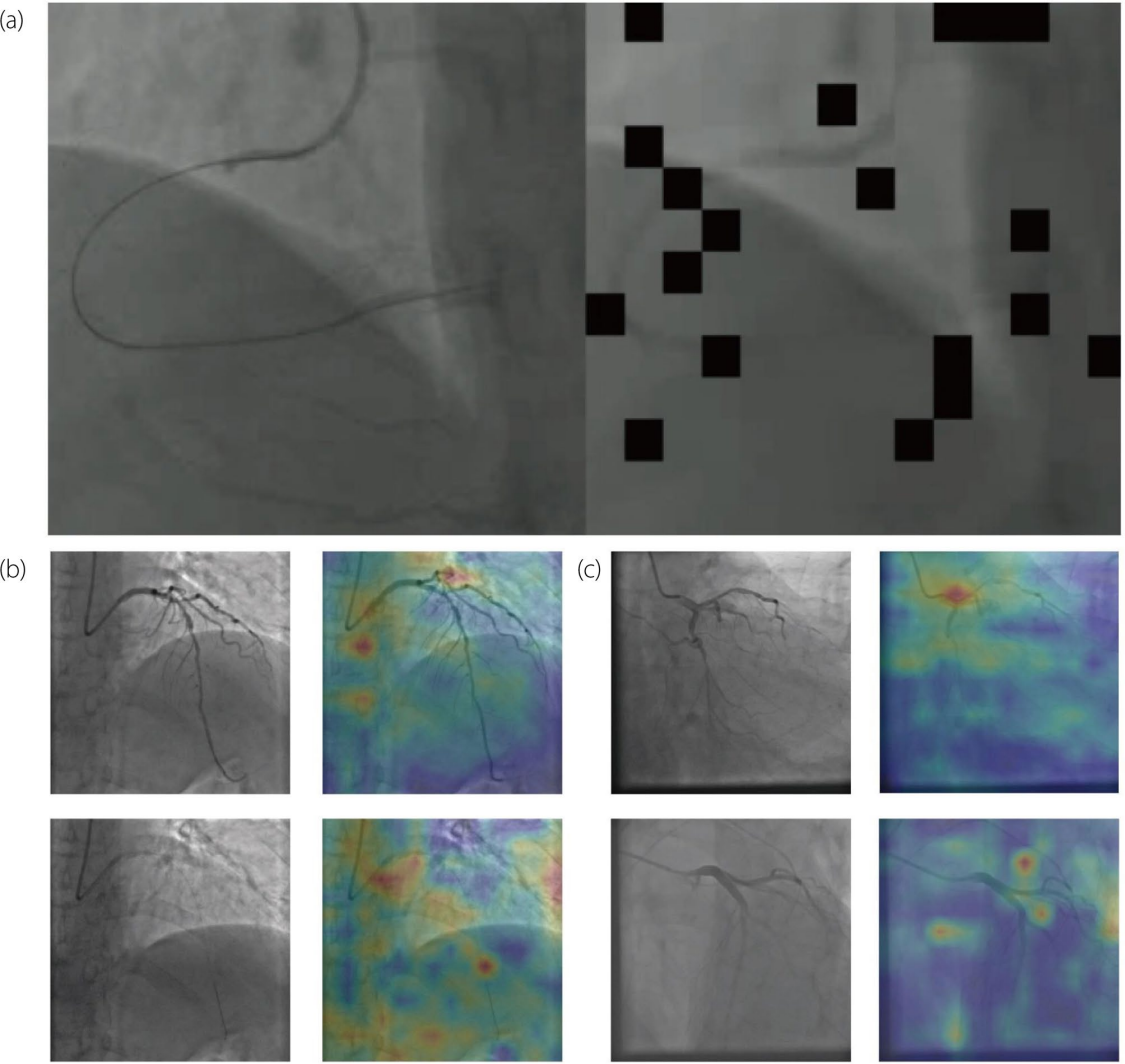
Model interpretation. **a** Corresponding pair of an input coronary angiography frame and the reconstructed frame; **b** Corresponding pairs of input frames from patients with severe aortic stenosis and heatmaps of highlighted regions contributing to the diagnosis (color intensity ranges from red for most important to green for less important regions); **c** Corresponding pairs of input frames from patients with severe mitral regurgitation and heatmaps of highlighted regions contributing to the diagnosis (color intensity ranges from red for most important to green for less important regions)

CAGFound demonstrated the ability to reconstruct major anatomical structures from CAG frames (Fig. 5a) and to generate complete angiographic videos (video in Additional File 2). This finding indicates that CAGFound learned disease-related features through the SSL process. To further investigate how CAGFound identified severe AS and severe MR, saliency heatmaps were generated to visualize the image regions most influential in classification decisions. For severe AS detection, the left atrium, aortic valve, and cardiac silhouette were highlighted as regions contributing to the diagnosis (Fig. 5b). For severe MR detection, the left atrium and mitral valve were highlighted as regions prioritizing important features for classification (Fig. 5c).

## Discussion

In this study, a video-based foundation model was developed using standard coronary angiograms to detect severe AS and severe MR. The principal findings are as follows: CAGFound accurately discriminated patients with two types of VHD, severe AS (AUROC 0.932) and severe MR (AUROC 0.933); the model also performed well in external validation at a separate medical center. CAGFound achieved robust detection of severe AS (AUROC 0.879) and severe MR (AUROC 0.896) in the external dataset. By transforming CAG into an opportunity for VHD screening, CAGFound has the potential to improve the detection rate of severe AS and severe MR, facilitate timely clinical management, and ultimately improve prognosis without prolonging procedure time or imposing additional burdens on patients or healthcare systems.

Despite the high prevalence of VHD and advances in treatment options, systematic screening programs remain limited. More than half of patients with VHD are undiagnosed [5, 9]. Artificial intelligence has been applied to complex medical data, such as radiologic images and electrocardiograms, to improve the diagnosis of VHD. Efforts have been made to detect AS from chest radiographs [27], to screen for AS and MR using electrocardiography [5], and to assess moderate or severe regurgitant VHD using electrocardiography [22]. CAD and VHD are the most prevalent cardiac disorders, and their coexistence is common [28]. Invasive CAG is recommended before aortic valve intervention in patients with a high or very high (> 50%) pretest probability of obstructive CAD and in patients with severe secondary MR and ventricular dysfunction [8]. Automated assessment of severe AS and severe MR during CAG is clinically beneficial for several reasons, including establishing the diagnosis and initiating appropriate treatment in patients with CAD and severe VHD. During CAG, left ventriculography can be performed to diagnose AS and MR and to quantify their severity. The key benefits of CAGFound include no additional cost, no extra contrast volume, no additional procedures, and complete automation, positioning it as a highly efficient augmentation tool. In this study, 10%–30% of patients in the test datasets were found to have kidney dysfunction consistent with chronic kidney disease stage 3 or worse. These patients may particularly benefit from CAGFound, which enables screening for severe AS and severe MR without additional contrast administration. Furthermore, about 25% of patients underwent TTE after angiography in both the internal and external datasets of AS and in the external cohort of MR. This temporal pattern suggests that valvular pathology may not have been recognized during the index procedure, thereby delaying the diagnosis of VHD. In the OxVALVE study, 50.8% of patients had VHD. In that cohort, 61.6% of patients had previously undergone CAG; however, only 4.9% were documented to have pre-existing VHD [9]. These findings indicate that patients may benefit from CAGFound by receiving automated, real-time screening for severe AS and severe MR, thereby reducing the risk of delayed diagnosis.

CAGFound demonstrated robust performance using standard coronary angiographic views across both internal and external datasets. Left cranial and right cranial projection views were used to develop the model, as these projections provide visualization of the mitral and aortic valves in fluoroscopic images and approximate the three-chamber and two-chamber views in echocardiography [29]. With the aid of artificial intelligence, noncoronary information can be extracted from angiography. CAG has been used to assess cardiac systolic function [18], calculate cardiac index, and detect right ventricular dysfunction [30]. However, few studies have focused on artificial intelligence-assisted screening for severe AS and severe MR using angiography. In the field of artificial intelligence-assisted disease detection, foundation models outperform traditional specialized models by offering superior generalizability, data efficiency, and scalability. While smaller, task-specific models can achieve strong performance on specific, identically distributed datasets, they frequently experience performance degradation when deployed across institutions because of domain shift. In addition, specialized models are narrowly optimized for a single classification task and require large annotated datasets to learn each new disease from scratch. In contrast, foundation models capture holistic anatomical and pathological representations, enabling rapid adaptation to new downstream diagnostic tasks through efficient few-shot learning or parameter-efficient fine-tuning. The development of foundation models for medical imaging has been constrained by limited dataset size [21, 31]. Model generalizability depends on the size and diversity of the training data [32]. In this study, this challenge was addressed by pretraining CAGFound on a large-scale dataset comprising 117,383 unlabeled CAG sequences from seven medical centers. Given the breadth and diversity of the data, model performance is expected to be more robust [21]. In the present study, the model was adapted to two downstream tasks: diagnosing severe AS and severe MR. CAGFound screened these two life-threatening VHD with high AUROC values. The model generalized well to the external validation datasets, achieving consistently high AUROC values. Multitask learning has been shown to alleviate overfitting and improve generalizability when the task of interest has a limited number of samples [20]. CAGFound employed a video-based architecture to incorporate rich motion and anatomical information, both of which are crucial for accurate VHD assessment. Video-based foundation models can track anatomical features, localize spatiotemporal patterns, and capture dynamic motion [33, 34], thereby facilitating improved understanding of life-threatening VHD from multiple perspectives. CAGFound was compared with VideoMAEv2 and Video Swin for severe AS and severe MR detection. CAGFound outperformed both models, further validating its effectiveness and generalizability for angiographic VHD screening.

While the technical performance of CAGFound is promising, its ultimate value depends on successful integration into clinical workflows and its ability to meaningfully influence physician decision-making and patient outcomes. Shortly after angiography, the model can generate inferences from CAG images and provide alerts for suspected severe AS and severe MR. Immediate awareness during the procedure enables real-time adjustments and prompts focused attention on potential valvular pathology. In addition, alerts can trigger recommendations for targeted follow-up echocardiography. This approach may reduce missed diagnoses and support tailored therapeutic strategies. However, several implementation challenges must be considered. First, introducing new technology into the catheterization laboratory should not slow procedures or distract operators. The inference speed of CAGFound is 30 samples per second, and its output does not mandate operator action. Second, false-positive results could desensitize clinicians to model recommendations. Therefore, CAGFound was calibrated using a high-specificity threshold to minimize unnecessary alerts. Furthermore, the model is well suited for cloud-based deployment. By hosting the core model on a centralized server, local clinics can access its diagnostic capabilities through a lightweight web-based interface, requiring only a standard computer and internet connection without the need for local GPU infrastructure.

The interpretability of the CAGFound model is essential for establishing trust in its diagnostic outputs. Left atrial enlargement, mitral annular dilation, and a higher prevalence of atrial fibrillation have been reported in patients with severe MR [35]. Severe AS is associated with findings such as left ventricular hypertrophy, pulmonary venous dilation, and aortic valve calcification [27]. These pathological changes result in abnormal cardiac anatomy and motion, which can be visualized and detected by a video-based model. Saliency heatmaps identified regions of interest that were critical for classification. For severe AS detection, the left atrium, aortic valve, and cardiac silhouette were highlighted. These findings are consistent with the pathophysiological mechanisms of AS [8, 36]. For severe MR detection, the left atrium and mitral valve were highlighted. These regions have also been emphasized in other artificial intelligence-assisted MR detection studies using TTE images [3, 22].

There are several limitations to the current study. First, the study design was retrospective rather than prospective or conducted in a real-world setting. Second, other VHD, such as aortic regurgitation, mitral stenosis, or tricuspid regurgitation, were not included in the downstream diagnostic tasks. Third, CAGFound is not currently capable of distinguishing between different severities of VHD. The architecture of CAGFound provides a versatile platform for expanding VHD assessment. Based on this foundation model, its application may be extended by incorporating labels for additional types and severities of VHD, followed by fine-tuning. These areas warrant further investigation in future studies. In an ideal clinical setting, comprehensive echocardiographic assessment is performed prior to angiography. However, real-world practice varies. Our model aims to address these inconsistencies by providing a universal, automated screening step during angiography. It is intended to function as a high-sensitivity trigger for further evaluation rather than as a definitive diagnostic tool.

## Conclusions

CAGFound, a video-based foundation model, was developed to screen for severe AS and severe MR from CAG videos. The model achieves high diagnostic performance for these VHD without requiring additional diagnostic equipment or extra contrast. Its effectiveness was further validated in external cohorts from a separate medical center. CAGFound enables automated assessment of severe AS and MR and supports broader clinical application of artificial intelligence in CAG-based imaging.

## Supplementary Information

Below is the link to the electronic supplementary material.

Supplementary Material 1

Supplementary Material 2

## Data Availability

The datasets of videos used to train and validate CAGFound are not publicly available because of ethical concerns, although medical images have been anonymized with all personally identifiable information removed. The data is available for use with the proper request with the corresponding author and with the approval of the ethical committee of Chinese PLA General Hospital.

## Code availability

Our code is publicly available at: https://github.com/ZmingShao/CAGFound.

## Abbreviations

CAD: Coronary heart disease
CAG: Coronary angiography
AS: Aortic stenosis
MR: Mitral regurgitation
AUROC: Area under the receiver operating characteristic curve
VHD: Valvular heart disease
TTE: Transthoracic echocardiography
SSL: Self-supervised learning
MLP: Multilayer perceptron
EMA: Exponential moving average
PPV: Positive predictive value
NPV: Negative predictive value
DCA: Decision curve analysis
ViT-base: Base vision transformer
STEMI: ST-segment elevation myocardial infarction
NSTEMI: Non-ST-segment elevation myocardial infarction
