# Supplementary material for: Foundation model for screening severe mitral regurgitation and severe aortic stenosis from coronary angiograms
Source: Vis Comput Ind Biomed Art. 2026 Jun 17;9:10. doi: 10.1186/s42492-026-00221-5 (PMC13275965; doi:10.1186/s42492-026-00221-5)
Supplement: Supplementary file 1 — Supplementary Material 1 [file 42492_2026_221_MOESM1_ESM.docx]

Additional file

Table S1. Baseline characteristics of the internal cohorts of patients with and without severe AS

|  | Patients with severe AS（n=119） | Patients without severe AS (n=239) | *P*  Value |
| --- | --- | --- | --- |
| Age, mean (SD), y | 68.42±9.86 | 63.85±10.51 | ＜0.001 |
| Gender, No. (%) | 0.007 | | |
| Female | 51（42.86%） | 67（28.03%） |  |
| Male | 68（57.14%） | 172（71.97%） |  |
| BMI | 25.53±4.26 | 26.14±2.97 | 0.119 |
| Coronary angiogram indication | ＜0.001 | | |
| Non-ACS, No. (%) | 95（79.83%） | 18（7.53%） |  |
| NSTEMI, No. (%) | 2（1.68%） | 7（2.93%） |  |
| STEMI, No. (%) | 0（0） | 19（7.95%） |  |
| Unstable angina, No. (%) | 22（18.49%） | 195（81.59%） |  |
| TTE Detail |  | | |
| Absolute time between TTE and coronary angiogram, mean (SD), d | 5.52±5.26 | 2.81±3.33 | ＜0.001 |
| Time between TTE and coronary angiogram, (%) |  | | 0.001 |
| 1-30d before angiogram, No. (%) | 103（86.55） | 183（76.57） | ＜0.001 |
| 0-14d after angiogram, No. (%) | 13（10.92） | 56（23.43） | ＜0.001 |
| 15-30d after angiogram, No. (%) | 3（2.52） | 0（0） | 0.083 |
| HBP | 75（63.03） | 181（75.73） | ＜0.001 |
| Scr | 100.38±102.89 | 91.71±82.10 | 0.389 |
| eGFR, ml/min/1.73m^2^ | 78.82±24.45 | 85.46±20.79 | 0.008 |
| eGFR |  | | 0.024 |
| eGFR>60, No. (%) | 95（79.83） | 212（88.70） | ＜0.001 |
| eGFR 45-60, No. (%) | 10（8.40） | 18（7.53） | 0.130 |
| eGFR 30-45, No. (%) | 7（5.82） | 3（1.25） | 0.205 |
| eGFR <30, No. (%) | 7（5.82） | 6（2.51） | 0.781 |

BMI, body mass index; ACS, acute coronary syndrome; NSTEMI, non-ST-segment elevation myocardial infarction; STEMI, ST-segment elevation myocardial infarction; TTE, transthoracic echocardiogram; HBP, high blood pressure; Scr, serum creatinine; eGFR, estimated glomerular filtration rate

Table S2. Baseline characteristics of the external cohorts of patients with and without severe AS

|  | Patients with severe AS（n= 44） | Patients without severe AS (n=88) | *P*  Value |
| --- | --- | --- | --- |
| Age, mean (SD), y | 67.34±10.48 | 61.99±10.43 | 0.006 |
| Gender, No. (%) |  |  | 0.032 |
| Female | 21（47.73） | 24（27.27） |  |
| Male | 23（52.27） | 64（72.73） |  |
| BMI | 22.96±4.05 | 25.73±3.20 | ＜0.001 |
| Coronary angiogram indication |  | | ＜0.001 |
| Non-ACS, No. (%) | 30（68.18） | 10（11.36） |  |
| NSTEMI, No. (%) | 0（0） | 4（4.55） |  |
| STEMI, No. (%) | 1（2.27） | 2（2.27） |  |
| Unstable angina, No. (%) | 13（29.55） | 72（81.82） |  |
| TTE Detail |  | | |
| Absolute time between TTE and coronary angiogram, mean (SD), d | 3.41±3.66 | 3.41±4.83 | 1.000 |
| Time between TTE and coronary angiogram, (%) |  | | ＜0.001 |
| 1-30d before angiogram, No. (%) | 0（0） | 49（55.68） | ＜0.001 |
| 0-14d after angiogram, No. (%) | 28（63.64） | 36（40.91） | 0.317 |
| 15-30d after angiogram, No. (%) | 16（36.36） | 3（3.41） | 0.002 |
| HBP | 26（59.09） | 62（70.45） | ＜0.001 |
| Scr | 82.24±30.31 | 84.92±24.96 | 0.590 |
| eGFR, ml/min/1.73m^2^ | 83.07±18.88 | 85.60±17.19 | 0.441 |
| eGFR |  |  | 0.522 |
| eGFR>60, No. (%) | 39（88.64） | 78（88.64） | ＜0.001 |
| eGFR 45-60, No. (%) | 2（4.55） | 6（6.82） | 0.157 |
| eGFR 30-45, No. (%) | 2（4.55） | 4（4.55） | 0.414 |
| eGFR <30, No. (%) | 1（2.27） | 0（0） | 0.317 |

BMI, body mass index; ACS, acute coronary syndrome; NSTEMI, non-ST-segment elevation myocardial infarction; STEMI, ST-segment elevation myocardial infarction; TTE, transthoracic echocardiogram; HBP, high blood pressure; Scr, serum creatinine; eGFR, estimated glomerular filtration rate

Table S3. Baseline characteristics of the internal cohorts of patients with and without severe MR

|  | Patients with severe MR（n=280） | Patients without severe MR (n=568) | *P*  Value |
| --- | --- | --- | --- |
| Age, mean (SD), y | 65.34±8.42 | 61.70±10.12 | ＜0.001 |
| Gender, No. (%) | ＜0.001 | | |
| Female | 143 (51.07) | 130 (22.89) |  |
| Male | 137 (48.93) | 438 (77.11) |  |
| BMI | 24.56±3.19 | 26.08±3.20 | ＜0.001 |
| Coronary angiogram indication | ＜0.001 | | |
| Non-ACS, No. (%) | 248 (88.57) | 34 (5.99) |  |
| NSTEMI, No. (%) | 1 (2.50) | 23 (4.05) |  |
| STEMI, No. (%) | 1 (2.50) | 10 (1.76) |  |
| Unstable angina, No. (%) | 30 (10.71) | 501 (88.20) |  |
| TTE Detail |  | | |
| Absolute time between TTE and coronary angiogram, mean (SD), d | 5.57±5.76 | 3.57±3.85 | <0.001 |
| Time between TTE and coronary angiogram, (%) |  | | <0.001 |
| 1-30d before angiogram, No. (%) | 239 (85.35) | 490 (86.27) | <0.001 |
| 0-14d after angiogram, No. (%) | 31 (11.07) | 10 (1.76) | 0.001 |
| 15-30d after angiogram, No. (%) | 10 (3.57) | 1 (0.18) | 0.006 |
| HBP | 110 (39.29) | 406 (71.48) | <0.001 |
| Scr | 90.77±81.17 | 84.46±53.71 | 0.178 |
| eGFR, ml/min/1.73m^2^ | 80.06±19.26 | 89.12±18.47 | ＜0.001 |
| eGFR |  | | 0.006 |
| eGFR>60, No. (%) | 243 (86.79) | 528 (92.96) | <0.001 |
| eGFR 45-60, No. (%) | 23 (8.21) | 27 (4.75) | 0.571 |
| eGFR 30-45, No. (%) | 10 (3.57) | 5 (0.88) | 0.196 |
| eGFR <30, No. (%) | 4 (1.43) | 8 (1.41) | 0.248 |

BMI, body mass index; ACS, acute coronary syndrome; NSTEMI, non-ST-segment elevation myocardial infarction; STEMI, ST-segment elevation myocardial infarction; TTE, transthoracic echocardiogram; HBP, high blood pressure; Scr, serum creatinine; eGFR, estimated glomerular filtration rate

Table S4. Baseline characteristics of the external cohorts of patients with and without severe MR

|  | Patients with severe MR（n=65） | Patients without severe MR (n=139) | *P*  Value |
| --- | --- | --- | --- |
| Age, mean (SD), y | 64.23±8.38 | 61.00±10.84 | 0.035 |
| Gender, No. (%) |  |  | 0.001 |
| Female | 32 (49.23) | 36 (25.90) |  |
| Male | 33 (50.77) | 103 (74.10) |  |
| BMI | 25.02±3.95 | 26.17±3.07 | 0.024 |
| Coronary angiogram indication | ＜0.001 | | |
| Non-ACS, No. (%) | 47 (72.31) | 15 (10.79) |  |
| NSTEMI, No. (%) | 5 (7.69) | 3 (2.16) |  |
| STEMI, No. (%) | 13 (20) | 3 (2.16) |  |
| Unstable angina, No. (%) | 0 (0) | 117 (84.17) |  |
| TTE Detail |  | | |
| Absolute time between TTE and coronary angiogram, mean (SD), d | 5.77±5.77 | 3.68±4.12 | 0.004 |
| Time between TTE and coronary angiogram, (%) |  | | 0.299 |
| 1-30d before angiogram, No. (%) | 50 (76.92) | 104 (74.82) | ＜0.001 |
| 0-14d after angiogram, No. (%) | 15 (23.08) | 30 (21.58) | 0.025 |
| 15-30d after angiogram, No. (%) | 0 (0) | 5 (3.60) | 0.025 |
| HBP | 30 (46.15) | 95 (68.35) | <0.001 |
| Scr | 100.76±66.71 | 88.08±54.58 | 0.154 |
| eGFR, ml/min/1.73m^2^ | 74.10±22.45 | 86.61±18.45 | <0.001 |
| eGFR>60, No. (%) | 46 (70.77) | 123 (88.49) | <0.001 |
| eGFR 45-60, No. (%) | 10 (15.38) | 8 (5.76) | 0.637 |
| eGFR 30-45, No. (%) | 7 (10.77) | 4 (2.88) | 0.365 |
| eGFR <30, No. (%) | 2 (3.08) | 1 (0.72) | 0.563 |

BMI, body mass index; ACS, acute coronary syndrome; NSTEMI, non-ST-segment elevation myocardial infarction; STEMI, ST-segment elevation myocardial infarction; TTE, transthoracic echocardiogram; HBP, high blood pressure; Scr, serum creatinine; eGFR, estimated glomerular filtration rate

Table S5. Comparison of different models on severe AS diagnosis

| Model | AUROC (95%CI) | Sensitivity (95%CI) | Specificity (95%CI) | PPV (95%CI) | NPV (95%CI) | ACC (95%CI) | F1 | Kappa |
| --- | --- | --- | --- | --- | --- | --- | --- | --- |
| CAGFound | 0.879  (0.809-0.948) | 0.800 (0.662-0.891) | 0.955 (0.890-0.982) | 0.900 (0.769-0.960) | 0.904 (0.827-0.949) | 0.903 (0.841-0.942) | 0.847 | 0.776 |
| VideoMAEv2 | 0.852 (0.776-0.927) | 0.756 (0.613-0.858) | 0.865 (0.779-0.921) | 0.739 (0.597-0.844) | 0.875 (0.790-0.929) | 0.828 (0.756-0.883) | 0.747 | 0.617 |
| Video Swin | 0.751 (0.659-0.844) | 0.578 (0.433-0.710) | 0.865 (0.779-0.921) | 0.684 (0.525-0.809) | 0.802 (0.711-0.869) | 0.769 (0.690-0.832) | 0.627 | 0.461 |

Abbreviations: MR, mitral regurgitation; AUROC, area under the receiver operating characteristic curve; PPV, positive predictive value; NPV, negative predictive value; ACC, accuracy

Table S6. Comparison of different models on severe MR diagnosis

| Model | AUROC (95%CI) | Sensitivity (95%CI) | Specificity (95%CI) | PPV (95%CI) | NPV (95%CI) | ACC (95%CI) | F1 | Kappa |
| --- | --- | --- | --- | --- | --- | --- | --- | --- |
| CAGFound | 0.896 (0.844-0.948) | 0.764 (0.640-0.840) | 0.855 (0.787-0.904) | 0.722 (0.610-0.812) | 0.874 (0.808-0.920) | 0.821 (0.763-0.867) | 0.738 | 0.602 |
| VideoMAEv2 | 0.832 (0.767-0.896) | 0.681 (0.564-0.779) | 0.790 (0.715-0.850) | 0.618 (0.506-0.719) | 0.832 (0.759-0.886) | 0.754 (0.691-0.807) | 0.648 | 0.459 |
| Video Swin | 0.830 (0.765-0.894) | 0.710 (0.594-0.804) | 0.783 (0.707-0.843) | 0.620 (0.510-0.719) | 0.844 (0.771-0.897) | 0.758 (0.696-0.812) | 0.662 | 0.476 |

Abbreviations: MR, mitral regurgitation; AUROC, area under the receiver operating characteristic curve; PPV, positive predictive value; NPV, negative predictive value; ACC, accuracy
